# What Drives Respondents to Seroepidemiological Surveys? Insights From COVID-19 and Implications for Future Pandemics

**DOI:** 10.2188/jea.JE20250088

**Published:** 2026-02-05

**Authors:** Sophearen Ith, Ryo Kinoshita, Sho Miyamoto, Yui Tomo, Takeshi Arashiro, Satoru Arai, Shoko Sakuraba, Jun Sugihara, Takaji Wakita, Tadaki Suzuki, Motoi Suzuki, Daisuke Yoneoka

**Affiliations:** 1 Japan Institute for Health Security, Tokyo, Japan; 2 Department of Global Environmental Health, Graduate School of Medicine, The University of Tokyo, Tokyo, Japan; 3 Ministry of Health, Labour, and Welfare of Japan, Tokyo, Japan

**Keywords:** COVID-19, non-respondent bias, seroepidemiological survey, pandemic

## Abstract

**Background:**

Non-random participation can undermine the representativeness of seroepidemiological surveys. Despite their critical role in estimating disease spread during pandemics, non-response bias and methods to correct it require further investigation. This study aimed to examine sociodemographic characteristics and coronavirus disease 2019 (COVID-19)-related factors influencing participation in a seroepidemiological survey.

**Methods:**

We analyzed data from a national COVID-19 seroepidemiological survey in Japan between December 2022 and March 2023. We performed multivariable logistic regression analyses to estimate adjusted odds ratios (aOR) and their confidence intervals (CIs) after variable selection with the Group Least Absolute Shrinkage and Selection Operator.

**Results:**

Among 6,091 participants, factors associated with higher odds of seroepidemiological surveys participation included being female (aOR 2.08; 95% CI, 1.25–3.47), living in larger households versus living alone (two: aOR 2.34; 95% CI, 1.20–4.55; four or above: aOR 2.05; 95% CI, 1.03–4.06), higher education levels versus junior high school education (high school: aOR 2.66; 95% CI, 1.06–6.15; junior colleges, technical colleges, vocational schools: aOR 5.51; 95% CI, 1.94–15.07; university and above: aOR 3.30; 95% CI, 1.26–7.98), and having a higher household income versus earning <2 million yen (2–4 million yen: aOR 3.32; 95% CI, 1.52–7.33; 4–6 million yen: aOR 2.73; 95% CI, 1.20–6.23, ≥6 million yen: aOR 4.51; 95% CI, 1.91–10.59). Lower seroepidemiological survey participation odds were observed in those hesitant or unwilling to vaccinate (aOR 0.16; 95% CI, 0.09–0.29) and those perceiving a higher COVID-19 positivity rate among close contacts (aOR 0.98; 95% CI, 0.98–0.99).

**Conclusion:**

Education, income, household size, sex, vaccination status, and perceived infection risk influenced seroepidemiological survey participation. The findings highlight the need to account for non-response bias using weighted methods like inverse probability weighting.

## INTRODUCTION

Seroepidemiological surveys are vital during pandemics, providing essential data to estimate disease spread, evaluate intervention effectiveness, and guide policy decisions.^1^^,^^2^ During the coronavirus disease 2019 (COVID-19) pandemic, many asymptomatic and mild cases went unreported in routine case tracking, making it difficult to assess disease burden and determine the actual case fatality rate.^3^ Consequently, seroepidemiological surveys have been widely used to estimate cumulative COVID-19 incidence, evaluate population immunity, and monitor both infection- and vaccine-induced seroprevalence.^4^^–^^6^

Despite their importance in guiding pandemic responses at the population level, seroepidemiological surveys are susceptible to biases.^7^^,^^8^ Even when participants are randomly selected, seroprevalence estimates may not accurately reflect the target population due to non-random participation.^7^ Without proper correction methods, the reliability of findings remains uncertain.^9^ Addressing non-response bias requires gathering data on non-respondents to assess the representativeness of participants.^7^ However, this process is challenging and resource-intensive, requiring substantial time, effort, and funding to follow up with non-respondents.

Previous studies have identified several sociodemographic factors influencing participation in health surveys, including sex, education, income, and health status.^9^^–^^11^ Individuals with lower socioeconomic status, lower education levels, or certain health conditions are often less likely to participate, creating data gaps that hinder the generalizability of findings.^9^^,^^11^ During pandemics like COVID-19, factors such as fear of infection and social restrictions likely exacerbate nonresponse rates, further complicating data collection.^12^^,^^13^ Although previous studies have examined factors contributing to nonresponse in population-based health surveys, limited studies have focused on nonresponse in seroepidemiological surveys, particularly during public health emergencies like COVID-19.^7^^,^^8^

In June 2020, Japan launched a series of large-scale national seroepidemiological surveys to estimate COVID-19 antibody seroprevalence across different regions.^14^^–^^17^ However, response rates declined over time, highlighting the need to identify factors influencing participation to improve data reliability and enhance future survey design.^18^^,^^19^ This study aimed to examine sociodemographic and COVID-19-related factors associated with participation in seroepidemiological surveys during the pandemic. In addition, the study discusses recommendations for data analysis and proposes strategies to ensure data representativeness, ultimately strengthening future survey methodologies and preparedness for emerging infectious diseases.

## METHODS

### Study design, setting, and participants

This cross-sectional study was part of Japan’s fifth national COVID-19 seroepidemiological survey, conducted from December 2022 to March 2023, in five prefectures: Miyagi, Tokyo, Aichi, Osaka, and Fukuoka (eFigure 1).^19^ The inclusion criteria for this study were: (1) being aged ≥20 years, (2) being residents of the selected municipalities (including both Japanese and foreign nationals), and (3) having received a mailed invitation and being able to provide informed consent. The study was approved by the Medical Research Ethics Committee of the National Institute of Infectious Diseases (NIID) and conducted in accordance with the Declaration of Helsinki (approval numbers 1457, 1472, and 1730). The survey, particularly for non-respondents (defined below), was carried out as a public health investigation under the Act on the Prevention of Infectious Diseases and Medical Care for Patients with Infectious Diseases (Infectious Disease Control Law) and was planned by the Ministry of Health, Labour, and Welfare (MHLW) and NIID.

### Sampling and data collection procedure

Using the Basic Resident Register, this study employed a multistage sampling method to select residents for mailed invitations. Within each prefecture, three municipalities were chosen to ensure representation from different municipality sizes: small (<100,000 population), medium (≥100,000 population), and large (ordinance-designated city/special ward). The study aimed to enroll 15,000 individuals (3,000 per prefecture). To achieve this, 75,000 residents (5,000 per municipality) were randomly selected, assuming a 20% response rate (eTable 1). Only one resident per household participated, and participants were informed they would receive their antibody titer results upon study completion.

After identifying potential participants in each municipality, we mailed research information, including a survey questionnaire and an invitation. Residents who wished to participate were instructed to visit the survey site with a completed questionnaire and written consent form and to provide a blood sample. At the site, each participant watched an explanatory video about the study. These individuals were classified as “Respondents” to the seroepidemiological survey. A flowchart illustrating the sampling procedure is shown in eFigure 2.

We classified “Non-respondents” to the seroepidemiological survey as residents who received an invitation but did not visit the survey site. A home-visit survey of non-respondents was conducted in Osaka city, where the response rate was the lowest among all locations. A list of non-respondents was created by cross-referencing registered invitees with respondents list. Subsequently, 400 households were randomly selected. Data collectors visited each selected household, explained the study’s purpose, and obtained informed consent before administering the same questionnaire.

Questionnaire data were entered into a database, and each participant was assigned an identification number to ensure anonymity and confidentiality. All personally identifiable information was removed before being shared with the research team.

### Variables and measurements

The survey utilized a structured questionnaire covering sociodemographic characteristics and COVID-19-related factors. Sociodemographic characteristics included eight items: study site, age, biological sex, comorbidities, household size, highest educational attainment, occupation, and annual household income. COVID-19-related information comprised nine items: vaccination status, household members’ infection history, perceived COVID-19 positivity and vaccination rates among regular contacts, exposure to a COVID-19-positive person without a mask for ≥15 minutes, preventive measures, history of COVID-19 infection, symptoms at diagnosis with COVID-19, and self-perceived immunity against COVID-19.

### Statistical analyses

Participants with incomplete data were excluded from the analysis. The analyses began with descriptive analyses to summarize the sociodemographic characteristics and COVID-19-related information of all participants, including respondents and non-respondents. To compare the characteristics between the two groups, the chi-square test was used for categorical variables, with Fisher’s exact test applied when any cell contained fewer than five observations. Student *t*-test was used for continuous variables. To identify factors associated with survey participation and estimate participation probability, multivariable logistic regression analyses were performed. Two models were developed for this purpose: model 1 included only sociodemographic variables to identify general factors influencing seroepidemiological survey respondents, aiming to minimize non-response bias in future pandemics beyond COVID-19; model 2 incorporated both sociodemographic and COVID-19-related factors to examine the specific determinants of seroepidemiological survey respondents during COVID-19 or similar pandemics. Variable selection for both models was conducted using the Least Absolute Shrinkage and Selection Operator (LASSO), which applies a group-level penalty to determine the inclusion or exclusion of related variable groups based on their relevance.^20^ This approach reduces the risk of overfitting by shrinking coefficients of irrelevant variables toward zero. Variable groups were defined based on dummy variables derived from categorical variables, such as education level, resulting in a total of 34 variables. Hyperparameters were tuned using a 10-fold cross-validation, and the coefficients of the selected variables were re-estimated. The significance level was set at 5%, with adjusted odds ratios (aORs), confidence intervals (CIs), and *P*-values reported. To assess the robustness of the results, a sensitivity analysis was conducted using data from Osaka city. We also assessed the impact of excluding participants with incomplete data by running the multivariable logistic regression models on the complete dataset (*n* = 8,316). All statistical analyses were performed using R (version 4.4.1; R Foundation for Statistical Computing, Vienna, Austria), with the *grpreg* (version 3.5.0) and *stats* (version 4.4.1) packages.

## RESULTS

### Sociodemographic characteristics

After excluding 2,384 participants with incomplete questionnaire responses, this study included a total of 6,091 participants, 98.6% of whom were the respondents to the seroepidemiological survey. The sociodemographic characteristics of the participants are presented in Table 1. The age distribution was as follows: 10.5% were aged 20–34 years, 29.7% were 35–49 years, 35.9% were 50–64 years, and 23.9% were ≥65 years. Among the participants, 60.3% were female, 12.6% lived alone, 40.4% had attained a university-level education or higher, and 30.2% were primarily engaged in office work. In addition, approximately one-third (35.5%) of participants had an annual household income exceeding 6 million yen.

**Table 1.** Comparison of sociodemographic characteristics and COVID-19-related factors between respondents and non-respondents in the seroepidemiological survey in Japan (*n* = 6,091)

Variable	Total (*N* = 6,091) *n* (%)	Non-respondents (*N* = 86, 1.4%) *n* (%)	Respondents (*N* = 6,005, 98.6%) *n* (%)	*P*-value^a^
Sociodemographic characteristics				
Age, years (SA)				
20–34	641 (10.5)	15 (17.4)	626 (10.4)	0.055
35–49	1,808 (29.7)	24 (27.9)	1,784 (29.7)	
50–64	2,184 (35.9)	22 (25.6)	2,162 (36.0)	
≥65	1,458 (23.9)	25 (29.1)	1,433 (23.9)	
Sex (SA)				
Male	2,417 (39.7)	41 (47.7)	2,376 (39.6)	0.157
Female	3,674 (60.3)	45 (52.3)	3,629 (60.4)	
Currently undergoing treatment or follow-up (comorbidities)				
Mean (SD)	0.4 (0.7)	3.0 (1.4)	3.7 (1.1)	<0.001
Median [IQR]	0.0 [0.0, 1.0]	3.0 [2.0, 4.0]	4.0 [3.0, 4.0]	
Household size (SA)				
One	770 (12.6)	21 (24.4)	749 (12.5)	0.006
Two	2,062 (33.9)	20 (23.3)	2,042 (34.0)	
Three	1,408 (23.1)	20 (23.3)	1,388 (23.1)	
Four and above	1,851 (30.4)	25 (29.1)	1,826 (30.4)	
Highest level of education attainment (SA)				
Junior high school	134 (2.2)	9 (10.5)	125 (2.1)	<0.001
High school/vocational school	1,927 (31.6)	32 (37.2)	1,895 (31.6)	
Junior colleges, technical colleges, and vocational schools	1,569 (25.8)	13 (15.1)	1,556 (25.9)	
University and above	2,461 (40.4)	32 (37.2)	2,429 (40.4)	
Occupation/Profession (SA)				
Mainly office work (including administration, planning, development, and government agency)	1,840 (30.2)	22 (25.6)	1,818 (30.3)	0.513
Mainly outside work (including sales, production, manufacturing, field, transportation, and retail)	1,107 (18.2)	20 (23.3)	1,087 (18.1)	
Medical care, Helper/Caregiver	672 (11.0)	12 (14.0)	660 (11.0)	
Education (including kindergartens, nurseries, elementary, junior high school, and students)	342 (5.6)	4 (4.7)	338 (5.6)	
Housewife and unemployed	1,523 (25.0)	23 (26.7)	1,500 (25.0)	
Others	607 (10.0)	5 (5.8)	602 (10.0)	
Annual household income (SA)				
Less than 2 million yen	539 (8.8)	17 (19.8)	522 (8.7)	<0.001
2–4 million yen	1,258 (20.7)	14 (16.3)	1,244 (20.7)	
4–6 million yen	1,053 (17.3)	14 (16.3)	1,039 (17.3)	
Over 6 million yen	2,160 (35.5)	16 (18.6)	2,144 (35.7)	
I don’t know/I don’t want to answer	1,081 (17.7)	25 (29.1)	1,056 (17.6)	
COVID-19 related variables				
Vaccination dose (SA)				
Mean (SD)	3.7 (1.1)	3.1 (1.4)	3.6 (1.1)	<0.001
Median [IQR]	4.0 [3.0, 4.0]	3.0 [2.0, 4.0]	4.0 [3.0, 4.0]	
Current vaccination status for available COVID-19 vaccines (SA)				
Fully vaccinated	3,485 (57.2)	24 (27.9)	3,461 (57.6)	<0.001
I haven’t been vaccinated yet, but I plan to get vaccinated	1,199 (19.7)	15 (17.4)	1,184 (19.7)	
Hesitant to get vaccinated/Not intending to get vaccinated	1,407 (23.1)	47 (54.7)	1,360 (22.6)	
Have at least one family member infected by COVID-19	1,797 (29.5)	28 (32.6)	1,769 (29.5)	0.612
Perceived COVID-19 positive rate (%) among regular contacts (SA)				
Mean (SD)	16.6 (20.8)	25.5 (27.7)	16.5 (20.6)	<0.001
Median [IQR]	10.0 [1.0, 25.0]	10.0 [0.2, 50.0]	10.0 [1.0, 25.0]	
Experience of being with a positive person for more than 15 minutes without a mask	1,199 (19.7)	21 (24.4)	1,178 (19.6)	0.329
Perceived COVID-19 vaccination rate (%) among regular contacts (SA)				
Mean (SD)	91.1 (14.1)	86.7 (16.4)	91.2 (14.1)	0.004
Median [IQR]	95.0 [90.0, 100.0]	90.0 [80.0, 100.0]	95.0 [90.0, 100.0]	
Preventive measures against COVID-19 infection (MA)				
Avoid places with poor ventilation	3,167 (52.0)	43 (50.0)	3,124 (52.0)	0.792
Avoid places where there are a lot of people.	3,599 (59.1)	50 (58.1)	3,549 (59.1)	0.945
Avoid talking or speaking in close proximity to other people	2,351 (38.6)	33 (38.4)	2,318 (38.6)	1
Washing your hands, gargling, and disinfecting your hands and fingers with alcohol	5,553 (91.2)	81 (94.2)	5,472 (91.1)	0.422
When coughing or sneezing, cover your mouth with a mask or handkerchief	4,649 (76.3)	54 (62.8)	4,595 (76.5)	0.004
I work remotely	311 (5.1)	6 (7.0)	305 (5.1)	0.584
Be mindful of social distancing	2,451 (40.2)	30 (34.9)	2,421 (40.3)	0.363
Avoid unnecessary going out	2,053 (33.7)	31 (36.0)	2,022 (33.7)	0.728
Others	271 (4.4)	2 (2.3)	269 (4.5)	0.592
There’s nothing in particular I’m doing	95 (1.6)	2 (2.3)	93 (1.5)	0.39
Have been infected with COVID-19 (SA)	1,166 (19.1)	27 (31.4)	1,139 (19.0)	0.006
Symptoms and signs when diagnosed as positive (MA) *n* = 1,166				
Severe fatigue	510 (43.7)	15 (55.5)	495 (43.7)	0.004
Chills	425 (36.4)	11 (40.7)	414 (36.3)	0.055
Joint pain	313 (26.8)	9 (33.3)	304 (26.7)	0.045
Headache	419 (35.9)	11 (40.7)	408 (35.8)	0.049
Runny nose	332 (28.5)	5 (18.5)	327 (28.7)	1
Cough	582 (49.9)	12 (44.4)	570 (50.0)	0.225
Sore throat	763 (65.4)	17 (62.9)	746 (65.5)	0.06
Shortness of breath	100 (8.6)	5 (18.5)	95 (8.3)	0.008
Nausea, diarrhea, abdominal pain	98 (8.4)	1 (3.7)	97 (8.5)	1
Pneumonia	6 (0.5)	0 (0.0)	6 (0.5)	1
Difficulty in sensing taste or smell	201 (17.2)	5 (18.5)	196 (17.2)	0.312
Others	250 (21.4)	2 (7.4)	248 (21.7)	0.585
No particular symptoms (normal condition)	43 (3.7)	2 (7.4)	41 (3.6)	0.123
Perception of own immunity against COVID-19 (SA)				
I think I have enough immunity	1,156 (19.0)	17 (19.8)	1,139 (19.0)	0.81
I had enough, but I’m worried my immunity is weakening	1,072 (17.6)	13 (15.1)	1,059 (17.6)	
I don’t think I have enough immunity	1,215 (19.9)	15 (17.4)	1,200 (20.0)	
I don’t know	2,648 (43.5)	41 (47.7)	2,607 (43.4)	

COVID-19, coronavirus disease 2019; IQR, interquartile range; MA, multiple-answer; SA, single-answer question; SD, standard deviation.

^a^The Student’s *t*-test was used for continuous variables, whereas the chi-square test or Fisher’s exact test (for cells with fewer than five observations) was used for categorical variables.

### COVID-19 related variables

As presented in Table 1, participants received the COVID-19 vaccine an average of 3.7 times, with 57.2% identifying as fully vaccinated and 23.1% expressing hesitancy or unwillingness to be vaccinated. Approximately 30% reported having at least one household member infected with COVID-19. On average, participants perceived that approximately 16% of their regular contacts had contracted COVID-19, while 90% were vaccinated. The majority (91.2%) practiced preventive measures, such as hand washing, gargling, and using alcohol-based hand disinfectants. In addition, 76.3% reported covering their mouths with a mask or handkerchief when coughing or sneezing, and 59.1% avoided crowded places. Approximately 20% of participants reported having been infected with COVID-19, with 43.7% experiencing severe fatigue, 65.4% suffering from a sore throat, and 49.9% developing a cough. Among all participants, 17.6% believed they had sufficient immunity against COVID-19 but were concerned about its decline.

### Factors associated with seroepidemiological survey participation

Table 2 presents the results of the logistic regression analysis identifying factors associated with participation in the seroepidemiological survey after variable selection using the Group LASSO approach. All sociodemographic variables assessed were assigned non-zero coefficients and were selected by the LASSO method. Several COVID-19 variables were assigned a coefficient of zero by the group LASSO and were not included in model 2. Model 1 revealed that the adjusted odds of participating in the seroepidemiological survey were statistically significantly higher among participants living in a two-member household (aOR 2.53; 95% CI, 1.33–4.85) than those living alone. Education was also a significant factor, with participants who attained high school/vocational school education (aOR 3.83; 95% CI, 1.63–8.23), junior college/technical college/vocational school education (aOR 7.62; 95% CI, 2.86–19.68), and university-level education or above (aOR 4.79; 95% CI, 1.96–10.82) having significantly higher odds of participation than those with a junior high school education. Household income was another determinant, as participants with an annual income of 2–4 million yen (aOR 2.89; 95% CI, 1.38–6.15), 4–6 million yen (aOR 2.32; 95% CI, 1.05–5.15), and over 6 million yen (aOR 3.76; 95% CI, 1.65–8.52) were significantly more likely to participate than those earning <2 million yen.

**Table 2.** Factor associated with respondents to the seroepidemiological survey (*n* = 6,091)

Variable	Model 1		Model 2	
	
	Respondents to the seroepidemiological survey aOR (95% CI)	*P*-value	Respondents to the seroepidemiological survey aOR (95% CI)	*P*-value
Sociodemographic characteristics				
Age, years (SA)				
20–34	Reference		Reference	
35–49	1.54 (0.77–3.01)	0.20	1.37 (0.67–2.74)	0.37
50–64	1.99 (0.97–3.99)	0.05	1.27 (0.60–2.64)	0.52
≥65	1.3 (0.57–2.96)	0.53	0.60 (0.25–1.46)	0.26
Sex (SA)				
Male	Reference		Reference	
Female	1.61 (1.00–2.61)	0.05	2.08 (1.25–3.47)	0.01
Currently undergoing treatment or follow-up (comorbidities)	1.12 (0.81–1.60)	0.52	1.08 (0.77–1.59)	0.65
Household size (SA)				
One	Reference		Reference	
Two	2.53 (1.33–4.85)	0.01	2.34 (1.20–4.55)	0.01
Three	1.66 (0.86–3.22)	0.13	1.55 (0.78–3.09)	0.20
Four and above	1.73 (0.90–3.31)	0.09	2.05 (1.03–4.06)	0.03
Highest level of education attainment (SA)				
Junior high school	Reference		Reference	
High school/vocational school	3.83 (1.63–8.23)	<0.001	2.66 (1.06–6.15)	0.02
Junior colleges, technical colleges, and vocational schools	7.62 (2.86–19.68)	<0.001	5.51 (1.94–15.07)	<0.001
University and above	4.79 (1.96–10.82)	<0.001	3.3 (1.26–7.98)	0.01
Occupation/Profession (SA)				
Mainly office work (including administration, planning, development, and government agency)	Reference		Reference	
Mainly outside work (including sales, production, manufacturing, field, transportation, and retail)	0.90 (0.47–1.73)	0.75	0.97 (0.50–1.89)	0.92
Medical care, Helper/Caregiver	0.59 (0.28–1.26)	0.15	0.50 (0.24–1.09)	0.07
Education (including kindergartens, nurseries, elementary, junior high school, and students)	1.21 (0.44–4.28)	0.73	1.23 (0.43–4.43)	0.72
Housewife and unemployed	1.27 (0.61–2.66)	0.51	1.28 (0.60–2.73)	0.52
Others	2.12 (0.82–6.61)	0.15	2.12 (0.81–6.67)	0.15
Annual household income (SA)				
Less than 2 million yen	Reference		Reference	
2 million yen or more but less than 4 million yen	2.89 (1.38–6.15)	0.01	3.32 (1.52–7.33)	0.01
Between 4 million and 6 million yen	2.32 (1.05–5.15)	0.03	2.73 (1.20–6.23)	0.01
Over 6 million yen	3.76 (1.65–8.52)	<0.001	4.51 (1.91–10.59)	<0.001
I don’t know/I don’t want to answer	1.07 (0.51–2.16)	0.86	1.20 (0.56–2.50)	0.62
COVID-19 related factors				
Current vaccination status for available COVID-19 vaccines (SA)	—	—		
Fully vaccinated	—	—	Reference	
I haven’t been vaccinated yet, but I plan to get vaccinated	—	—	0.52 (0.27–1.04)	0.05
Hesitant to get vaccinated/Not intending to get vaccinated	—	—	0.16 (0.09–0.29)	<0.001
Perceived COVID-19 positive rate (%) among regular contacts (SA)	—	—	0.98 (0.98–0.99)	<0.001
Perceived COVID-19 vaccination rate (%) among regular contacts	—	—	1.00 (0.99–1.02)	0.46
Preventive measures against COVID-19 infection (MA)	—	—		
Washing your hands, gargling, and disinfecting your hands and fingers with alcohol	—	—		
No	—	—	Reference	
Yes	—	—	0.36 (0.12–0.85)	0.03
When coughing or sneezing, cover your mouth with a mask or handkerchief	—	—		
No	—	—	Reference	
Yes	—	—	1.82 (1.12–2.90)	0.01
Avoid unnecessary going out	—	—		
No	—	—	Reference	
Yes	—	—	0.82 (0.50–1.35)	0.42
Symptoms and signs when diagnosed as positive (MA)	—	—		
Severe fatigue	—	—		
No	—	—	Reference	
Yes	—	—	0.62 (0.32–1.27)	0.16
Shortness of breath	—	—		
No	—	—	Reference	
Yes	—	—	0.56 (0.20–1.83)	0.28
Others	—	—		
No	—	—	Reference	
Yes	—	—	2.49 (0.75–15.50)	0.21
No particular symptoms (normal condition)	—	—		
No	—	—	Reference	
Yes	—	—	0.27 (0.07–1.83)	0.10

aOR, adjusted odds ratio; CI, confidence interval; COVID-19, coronavirus disease 2019; MA, multiple-answer; SA, single-answer question.

Model 2 examined factors influencing seroepidemiological survey participation in the context of the COVID-19 pandemic. Female participants had significantly higher odds of participation (aOR 2.08; 95% CI, 1.25–3.47) than males. Household size remained a factor, with individuals from two-member households (aOR 2.34; 95% CI, 1.20–4.55) and those in households with four or more members (aOR 2.05; 95% CI, 1.03–4.06) being more likely to participate than those living alone. Similar to model 1, individuals having a high school/vocational school education (aOR 2.66; 95% CI, 1.06–6.15), junior college/technical college/vocational school education (aOR 5.51; 95% CI, 1.94–15.07), and university-level education or above (aOR 3.30; 95% CI, 1.26–7.98) showed increased participation compared to those with a junior high school education. Higher annual household income was also a significant factor, with those earning 2–4 million yen (aOR 3.32; 95% CI, 1.52–7.33), 4–6 million yen (aOR 2.73; 95% CI, 1.20–6.23), and >6 million yen (aOR 4.51; 95% CI, 1.91–10.59) demonstrating greater participation than those earning <2 million yen. COVID-19-related factors also influenced seroepidemiological survey participation. Those hesitant or unwilling to get vaccinated were significantly less likely to participate (aOR 0.16; 95% CI, 0.09–0.29) compared to fully vaccinated individuals. In addition, a higher perceived COVID-19 positivity rate among regular contacts was associated with lower participation (aOR 0.98; 95% CI, 0.98–0.99). Preventive measures played a role, as individuals who covered their mouths with a mask or handkerchief when coughing or sneezing were more likely to participate (aOR 1.82; 95% CI, 1.12–2.90), whereas those who regularly practiced hand washing, gargling, and hand disinfection with alcohol were less likely to participate (aOR 0.36; 95% CI, 0.12–0.85) compared to those who did not engage in these practices.

A graph comparing the proportion of statistically significant factors between respondents and non-respondents in the seroepidemiological survey is presented in eFigure 3.

### Sensitivity analyses

The sensitivity analysis, conducted using the same models with a subset of data from Osaka city, revealed similar sociodemographic and COVID-19-related factors significantly associated with participation in the seroepidemiological survey. In model 1, some notable differences emerged. Age was a significant factor, with participants aged 35–49 years (aOR 2.74; 95% CI, 1.10–6.81) and 50–64 years (aOR 3.25; 95% CI, 1.29–8.26) showing significantly higher odds of participation than those aged 20–34 years. However, for participants aged ≥65 years, the association was not statistically significant (aOR 1.07; 95% CI, 0.36–3.16). In addition, female participants were significantly more likely to participate than males (aOR 2.06; 95% CI, 1.11–3.87). In model 2, the overall findings remained robust, with one key exception: participants employed in medical care, as helpers, or as caregivers (aOR 0.28; 95% CI, 0.10–0.79) had significantly lower odds of participation in the seroepidemiological survey. Unlike in the primary analysis, education level and perceived COVID-19 positivity among regular contacts were not statistically significant predictors of participation in this subset. The results of the sensitivity analyses are presented in eTable 2. Likewise, the sensitivity analysis of complete data also yielded similar sociodemographic and COVID-19-related factors significantly associated with survey participation (eTable 3).

## DISCUSSION

This study is among the few to identify factors influencing participation in a seroepidemiological survey, addressing the often-overlooked issue of non-response bias and providing insights to improve data-driven decision-making during pandemics. Our findings are relevant not only to the COVID-19 pandemic but also to future pandemics through two distinct models: model 1 offers a framework to mitigate non-response challenges in unknown pandemics, whereas model 2 provides targeted recommendations to address specific barriers observed during COVID-19 or similar pandemics. The seroepidemiological survey data were collected from five major prefectures, representing over 30% of Japan’s total population, thereby enhancing the relevance of the findings for a considerable portion of the population.^21^ In addition, the use of a random-sampling method may help reduce selection bias and contribute to the validity of the results. In particular, our survey uniquely incorporated home visits to follow up with non-respondents—an essential approach to reducing volunteer bias. This method is often challenging in seroepidemiological studies due to the substantial demands on time, resources, and logistical planning.^7^ Drawing on the collected data and analysis, sociodemographic factors associated with participation in the seroepidemiological survey included the highest level of educational attainment, annual household income, household size, and sex. COVID-19-related factors included vaccination status, perceived COVID-19 positivity rates among regular contacts, and adherence to specific preventive measures.

Both models indicated that participants with higher education levels or annual household income had greater odds of participating in the seroepidemiological survey than those with only a junior high school education or an annual household income of <2 million yen. Although direct comparisons with other studies are challenging due to differences in survey objectives, study designs, target populations, and data collection methods, previous studies have reported a similar trend—individuals with lower education and income levels are less likely to participate in health surveys.^22^ This may be because higher education often correlates with greater awareness of and interest in public health studies.^23^ Similarly, higher household income may provide more resources and flexibility to engage in surveys. Furthermore, this study did not offer financial compensation; instead, the sole incentive for participation was the return of anti-S and anti-N antibody titers. Consequently, individuals with lower incomes may have been less motivated to participate in the absence of monetary incentives. In addition, we observed that respondents living in two-member or four-or-more-member households had higher odds of participation than those living alone. A possible explanation is that individuals in smaller households may face fewer logistical challenges when participating in surveys.^9^^,^^24^ Furthermore, this may be partly explained by the higher proportion of single-person households in Osaka city, where individuals living alone may be less likely to participate. Consistent with previous studies,^9^^,^^11^ we found that females were more likely to participate in the survey than males. This could be attributed to conventional expectations of women in managing family health or a greater interest in health-related surveys.

Regarding COVID-19-related factors, participants who were hesitant or unwilling to be vaccinated were less likely to participate than those who were fully vaccinated. This may be because fully vaccinated individuals tend to have greater confidence in the vaccine’s protective effects, reducing their fear of infection and encouraging survey participation.^25^^,^^26^ However, further study is needed to investigate why individuals who are unwilling to be vaccinated were less likely to participate as other factors, including a lack of interest in knowing their antibody status, distrust in vaccines or immunology, or unwillingness to engage with COVID-19-related matters, could have also contributed to their lower participation. These findings suggest that careful consideration is needed when using collected data to assess the impact of vaccines on seroprevalence, particularly for S-antibodies, as results may overestimate vaccine effectiveness within the population.

In addition, participants who perceived a higher rate of COVID-19 positivity among their regular contacts were less likely to participate in the survey. This may stem from a fear of infection, as they might have avoided participation due to perceived risks associated with visiting survey sites or interacting with survey staff.^13^^,^^27^ Such decision is particularly understandable given that the survey was conducted during Japan’s 8th wave of COVID-19, which was largely driven by Omicron subvariants. During this period, the Japanese government announced in October 2022 that mask-wearing was generally unnecessary in outdoor settings and later decided in February 2023 to leave mask usage to individual discretion beginning March 13th.^28^ Although these were non-binding recommendations, adherence remained high due to strong cultural norms.^29^ National survey data further indicated that daily mask-wearing duration did not substantially change following vaccine uptake or policy shifts, and population contact rates remained well below pre-pandemic levels,^30^ likely contributing to reduced social interaction and survey participation.

Notably, individuals who adhered to preventive measures, such as wearing masks or covering their mouths with handkerchiefs, were more likely to participate, perhaps reflecting both their confidence in these precautions and a deeper commitment to communal well-being. Practicing cough etiquette can be conceptualized as an altruistic behavior, reflecting a heightened sense of prosocial motivation and voluntary behavior.^31^ Individuals who consistently engage in such behaviors may possess stronger concern for the well-being of others, which in turn may predispose them to participate in seroepidemiological surveys, particularly those designed to inform and protect public health efforts during infectious disease outbreaks. This interpretation offers a psychological perspective on the behavioral characteristics of respondents and may partly explain the association observed between cough etiquette and survey participation. Conversely, individuals who reported practicing preventive measures, such as washing hands, gargling, and disinfecting hands with alcohol, were also less likely to participate. This could be because those who are highly cautious about hygiene may have a stronger fear of exposure to COVID-19.^32^ Consequently, they might avoid environments or activities perceived as risky, such as surveys requiring physical presence or interaction. However, further research is needed to explore the reasons behind these differing behaviors.

These findings underscore the need for caution when interpreting collected seroepidemiological data and offer insights for designing future surveys to estimate the seroprevalence of infectious diseases. Given the differences between respondents and non-respondents, careful interpretation of the data is essential. For instance, serum anti-S values derived from vaccination may be overestimated due to higher vaccination rates among participants. To ensure representativeness, adjustments for non-response bias should be considered when analyzing collected data. Various statistical techniques, including weighting adjustments, can be applied to account for this bias.^33^ One practical approach is to use inverse probability weighting (IPW) by applying the estimated regression coefficients from our study. We propose two practical models: model 1 for future unknown pandemics and model 2 for COVID-19 or similar pandemics. Specifically, we recommend using the following survey weights, $w_{i}=1/P(\text{Participation}\;|\;x_{i})$ , for the *i*th individual: $P(\text{Participation}\;|\;x_{i})=\frac{1}{1+\exp(-x_{i}^{T}\hat{\beta }-\hat{\alpha })}$ , where ***x****_i_* represents the covariate vector for the *i*th individual, $\hat{\beta }$ is the vector of estimated regression coefficients in model 1 or model 2 (calculated as log[AOR]), and $\hat{\alpha }$ is the estimated intercept term ( $\hat{\alpha }=0.72$ ; for model 1 and $\hat{\alpha }=2.57$ for model 2). To provide more nuanced insights into the survey results, subgroup analyses should be conducted to explore variations across demographic groups, including income, sex, education level, and vaccination status.

Future seroepidemiological studies should incorporate survey designs that actively minimize participation barriers, such as home-based blood collection methods, particularly for individuals with a high perceived risk of infection.^34^ Broader sociodemographic coverage should also be prioritized to ensure the inclusion of diverse population groups. For instance, targeted recruitment efforts should focus on underrepresented groups, such as individuals with lower education levels or lower household incomes. Moreover, mailed survey invitations should emphasize the safety, importance, and robustness of infection control measures to address fears, dispel misconceptions, and boost participation, particularly among vaccine-hesitant individuals and those concerned about COVID-19 risks.

This study has some limitations worth noting. First, by restricting the sample to individuals aged ≥20 years, the findings may not be generalizable to younger populations, including adolescents and children. Second, although this study included multiple large prefectures with diverse demographic and geographic characteristics, the selection was not nationally comprehensive. While we attempted to enhance local representativeness by selecting three municipalities of different sizes within each participating prefecture, this strategy only ensures representativeness within the selected municipalities. These municipalities themselves may not fully reflect the diversity of all regions in Japan. In addition, future studies should consider using multilevel modeling with random effects to account for prefecture-specific differences. Therefore, while the findings provide important insights into participation dynamics, they should be interpreted with caution when generalizing to the broader Japanese population. Third, non-respondent data collection was limited to Osaka city, and the relatively small sample size may have constrained the ability to accurately assess differences in participation, potentially introducing bias and limiting generalizability. Although a sensitivity analysis for Osaka city was conducted to evaluate the robustness of the models, the result may reflect attributes of Osaka city population rather than true differences between respondents and non-respondents. This limitation should be taken into account when interpreting our findings. Future studies should aim to include a broader range of prefectures to improve generalizability. Fourth, this study did not include residents who received mailed invitations but did not visit the blood collection sites or participate in home visit surveys, which may have resulted in an underrepresentation of non-respondents. Fifth, reliance on self-reported data may have introduced recall bias. Additionally, a substantial number of participants with missing data were excluded. Although missing values were presumed to be unrelated to participant characteristics, the assumption that missing values occurred completely at random cannot be conclusively verified. If specific subgroups were more likely to have missing data, their exclusion could have led to underrepresentation, potentially affecting the generalizability of our findings.

In conclusion, this study identified several sociodemographic and COVID-19-related factors influencing participation in seroepidemiological surveys conducted during the pandemic. Being male, having lower education and income levels, living alone, vaccine hesitancy, adherence to certain preventive measures, and perceiving a higher COVID-19 positivity rate among regular contacts were all associated with a lower probability of participation. These findings highlight the importance of using IPW to reduce non-response bias in seroepidemiological surveys conducted during COVID-19 and future pandemics. Our study underscores the need for careful interpretation of survey data and provides valuable insights for designing future seroepidemiological surveys, ensuring more representative and reliable data, and contributing to future pandemic preparedness plans.

## SUPPLEMENTARY MATERIAL

eTable 1. Summary of the number of respondents who participated in the seroepidemiological survey included in this study

eTable 2. Sensitivity analysis of factors associated with seroepidemiological survey participation in Osaka City (n = 345)

eTable 3. Sensitivity analysis of factors associated with seroepidemiological survey participation without excluding incomplete data

eFigure 1. Map of the study area

eFigure 2. Flowchart illustrating the sampling procedure for respondents (6,005) and nonrespondents (86) of seroepidemiological survey

eFigure 3. Bar graph comparing the proportion of statistically significant factors among respondents and non-respondents in the seroepidemiological survey
