# Supplementary material for: What Drives Respondents to Seroepidemiological Surveys? Insights From COVID-19 and Implications for Future Pandemics
Source: J Epidemiol. 2026 Feb 5;36(2):58–66. doi: 10.2188/jea.JE20250088 (PMC12783504; doi:10.2188/jea.JE20250088)
Supplement: Supplementary file 1 [file je-36-058-s001.pdf]

eTable 1. Summary of the number of respondents who participated in the seroepidemiological survey included in this study

Prefecture	Expected number of participants	Distributed invitation mail	Number of residents participating in seroepidemiological survey	Number of "Respondents" included in the analysis
Miyagi	3,000	15,000	2,040	1,325
Tokyo	3,000	15,000	1,798	1,212
Aichi	3,000	15,000	1,427	1,160
Osaka	3,000	15,000	1,525	1,070
Fukuoka	3,000	15,000	1,367	1,238
Total	15,000	75,000	8,157	6,005

eTable 2. Sensitivity analysis of factors associated with seroepidemiological survey participation in Osaka City (n=345)

Variable	Model 1		Model 2	
	Respondents to the seroepidemiological survey aOR (95% CI)	*P*-value	Respondents to the seroepidemiological survey aOR (95% CI)	*P*-value
**Socio-economic characteristics**				
Age, years (SA)				
20–34	Reference		Reference	
35–49	2.74 (1.1–6.81)	0.029	2.27 (0.82–6.28)	0.113
50–64	3.25 (1.29–8.26)	0.012	2.04 (0.72–5.82)	0.18
≥65	1.07 (0.36–3.16)	0.909	0.52 (0.15–1.78)	0.303
Sex (SA)				
Male	Reference		Reference	
Female	2.06 (1.11–3.87)	0.023	2.53 (1.27–5.16)	0.009
Currently undergoing treatment or follow-up (comorbidities)	1.03 (0.68–1.62)	0.882	0.97 (0.61–1.59)	0.896
Household size (SA)				
One	Reference		Reference	
Two	1.67 (0.74–3.77)	0.216	1.50 (0.61–3.65)	0.374
Three	0.95 (0.4–2.27)	0.911	0.84 (0.31–2.25)	0.731
Four and above	0.96 (0.41–2.23)	0.927	1.23 (0.47–3.19)	0.67
Highest level of education attainment (SA)				
Junior high school	Reference		Reference	
High school/vocational school	2.72 (0.78–10.3)	0.123	2.36 (0.58–10.45)	0.239
Junior colleges, technical colleges, vocational schools	4.41 (1.14–18.69)	0.035	3.34 (0.74–16.47)	0.123
University and above	4.11 (1.13–16.31)	0.036	2.76 (0.64–12.81)	0.179
Occupation/Profession (SA)				
Mainly office work (eg, administration, planning, development, government agency)	Reference		Reference	
Mainly outside work (eg, sales, production, manufacturing, field, transportation, retail)	0.75 (0.33–1.68)	0.476	0.66 (0.27–1.63)	0.358
Medical care, Helper/Caregiver	0.41 (0.17–1.03)	0.053	0.28 (0.10–0.79)	0.015
Education (including kindergartens, nurseries, elementary, junior high school, and students)	0.8 (0.19–3.89)	0.768	0.52 (0.10–2.85)	0.426
Housewife and unemployed	1.37 (0.54–3.53)	0.513	0.98 (0.34–2.88)	0.974
Others	1.08 (0.36–3.76)	0.902	0.78 (0.23–3.01)	0.698
Annual household income (SA)				
Less than 2 million yen	Reference		Reference	
2 million yen or more but less than 4 million yen	3.09 (1.15–8.45)	0.026	3.93 (1.34–11.78)	0.013
Between 4 million and 6 million yen	2.3 (0.82–6.57)	0.114	3.23 (1.01–10.52)	0.049
Over 6 million yen	2.65 (0.91–7.7)	0.073	4.20 (1.26–14.15)	0.019
I don’t know/I don’t want to answer	0.72 (0.27–1.86)	0.501	1.16 (0.38–3.46)	0.795
**COVID-19 related factors**				
Current vaccination status for available COVID-19 vaccines (SA)	-	-		
Fully vaccinated	-	-	Reference	

I haven't been vaccinated yet, but I plan to get vaccinated	-	-	0.88 (0.37–2.11)	0.763
Hesitant to get vaccinated/Not intending to get vaccinated	-	-	0.21 (0.10–0.45)	<0.001
Perceived COVID-19 positive rate (%) among regular contacts (SA)	-	-	0.99 (0.98–1.01)	0.346
Perceived COVID-19 vaccination rate (%) among regular contacts	-	-	1.00 (0.98–1.02)	0.814
Preventive measures against COVID-19 infection (MA)	-	-		
Washing your hands, gargling, and disinfecting your hands and fingers with alcohol	-	-		
No	-	-	Reference	
Yes	-	-	0.20 (0.05–0.66)	0.014
When coughing or sneezing, cover your mouth with a mask or handkerchief	-	-		
No	-	-	Reference	
Yes	-	-	3.33 (1.67–6.76)	0.001
Avoid unnecessary going out	-	-		
No	-	-	Reference	
Yes	-	-	0.84 (0.43–1.65)	0.61
Symptoms and signs when diagnosed as positive (MA)	-	-		
Severe fatigue	-	-		
No	-	-	Reference	
Yes	-	-	0.46 (0.16–1.29)	0.134
Shortness of breath	-	-		
No	-	-	Reference	
Yes	-	-	1.09 (0.20–6.15)	0.924
Others	-	-		
No	-	-	Reference	
Yes	-	-	6.00 (1.36–43.75)	0.035
No particular symptoms (normal condition)	-	-		
No	-	-	Reference	
Yes	-	-	0.17 (0–4.40)	0.301

aOR, adjusted odds ratio; CI, confidence interval; COVID-19, coronavirus disease 2019; MA, multiple-answer; SA, single-answer question.

eTable 3. Sensitivity analysis of factors associated with seroepidemiological survey participation without excluding incomplete data

Variable	Model 1 (n=7,828)		Model 2 (n=7,286)	
	**Respondents to the health survey aOR (95% CI)**	***P*-value**	**Respondents to the health survey aOR (95% CI)**	***P*-value**
**Socio-economic characteristics**				
**Age, years (SA)**				
20–34	Reference		Reference	
35–49	1.88 (1.08–3.24)	0.02	1.73 (0.94–3.13)	0.07
50–64	2.43 (1.39–4.18)	<0.001	1.68 (0.89–3.10)	0.1
≥65	1.84 (0.96–3.50)	0.06	0.93 (0.43–1.98)	0.85
**Sex (SA)**				
Male	Reference		Reference	
Female	2.19 (1.51–3.20)	<0.001	2.60 (1.68–4.03)	<0.001
**Currently undergoing treatment or follow-up (comorbidities)**	1.14 (0.88–1.51)	0.33	1.10 (0.82–1.53)	0.53
**Household size (SA)**				
One	Reference		Reference	
Two	2.42 (1.50–3.91)	<0.001	2.77 (1.60–4.84)	<0.001
Three	2.13 (1.28–3.56)	<0.001	2.18 (1.23–3.89)	0.01
Four and above	2.55 (1.52–4.28)	<0.001	3.13 (1.75–5.61)	<0.001
**Highest level of education attainment (SA)**				
Junior high school	Reference		Reference	
High school/vocational school	2.49 (1.22–4.72)	0.01	2.38 (1.03–5.07)	0.03
Junior colleges, technical colleges, vocational schools	5.46 (2.40–11.98)	<0.001	4.92 (1.91–12.13)	<0.001
University and above	4.43 (2.07–8.96)	<0.001	3.54 (1.47–7.95)	<0.001
**Occupation/Profession (SA)**				
Mainly office work (eg, administration, planning, development, government agency)	Reference		Reference	
Mainly outside work (eg, sales, production, manufacturing, field, transportation, retail)	0.89 (0.53–1.50)	0.67	0.9 (0.51–1.59)	0.71
Medical care, helper/caregiver	0.62 (0.34–1.19)	0.14	0.65 (0.33–1.38)	0.24
Education (including kindergartens, nurseries, elementary, junior high school, and students)	0.94 (0.42–2.38)	0.88	1.27 (0.50–3.94)	0.65
Housewife and unemployed	1.21 (0.67–2.17)	0.53	1.12 (0.59–2.13)	0.74
Others	1.28 (0.66–2.62)	0.49	1.59 (0.74–3.74)	0.26
**Annual household income (SA)**				
Less than 2 million yen	Reference		Reference	
2 million yen or more but less than 4 million yen	2.08 (1.10–3.95)	0.02	2.89 (1.41–5.95)	<0.001

Between 4 million and 6 million yen	1.64 (0.82–3.29)	0.16	2.47 (1.15–5.37)	0.02
Over 6 million yen	2.63 (1.27–5.44)	0.01	3.68 (1.66–8.16)	<0.001
I don't know/I don't want to answer	0.67 (0.37–1.16)	0.16	0.90 (0.47–1.67)	0.75
**COVID-19 related factors**				
**Current vaccination status for available COVID-19 vaccines (SA)**	–	–	–	-
Fully vaccinated	–	–	Reference	
I haven't been vaccinated yet, but I plan to get vaccinated	–	–	0.64 (0.36–1.17)	0.14
Hesitant to get vaccinated/Not intending to get vaccinated	–	–	0.22 (0.13–0.35)	<0.001
**Perceived COVID-19 positive rate (%) among regular contacts (SA)**	–	–	0.98 (0.98–0.99)	<0.001
**Perceived COVID-19 vaccination rate (%) among regular contacts**	–	–	1.01 (1.00–1.02)	0.14
**Preventive measures against COVID-19 infection (MA)**	–	–		
Washing your hands, gargling, and disinfecting your hands and fingers with alcohol	–	–		
No	–	–	Reference	
Yes	–	–	0.48 (0.21–0.96)	0.06
When coughing or sneezing, cover your mouth with a mask or handkerchief	–	–		
No	–	–	Reference	
Yes	–	–	1.56 (1.02–2.34)	0.04
Avoid unnecessary going out	–	–	–	-
No	–	–	Reference	
Yes	–	–	0.83 (0.55–1.29)	0.4
**Symptoms and signs when diagnosed as positive**	–	–	–	-
Severe fatigue	–	–	–	-
No	–	–	Reference	
Yes	–	–	0.53 (0.30–0.98)	0.03
Shortness of breath	–	–	–	-
No	–	–	Reference	
Yes	–	–	0.86 (0.33–2.73)	0.77
others	–	–	–	-
No	–	–	Reference	
Yes	–	–	1.42 (0.60–4.30)	0.47
No particular symptoms (normal condition)	–	–	–	-
No	–	–	Reference	-
Yes	–	–	0.32 (0.08–2.17)	0.15

aOR, adjusted odds ratio; CI, confidence interval; COVID-19, coronavirus disease 2019; MA, multiple-answer; SA, single-answer question.

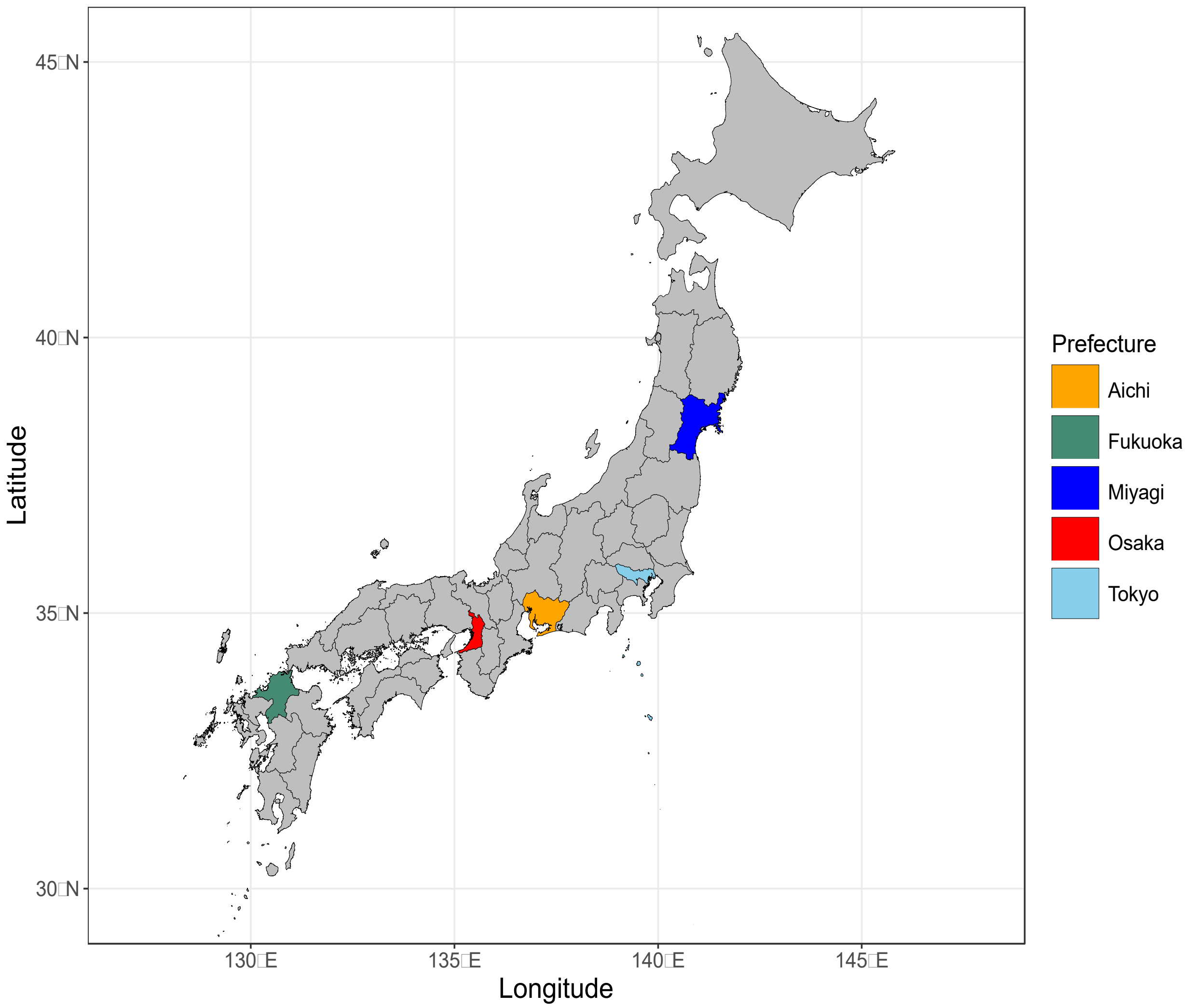

eFigure 1. Map of the study area

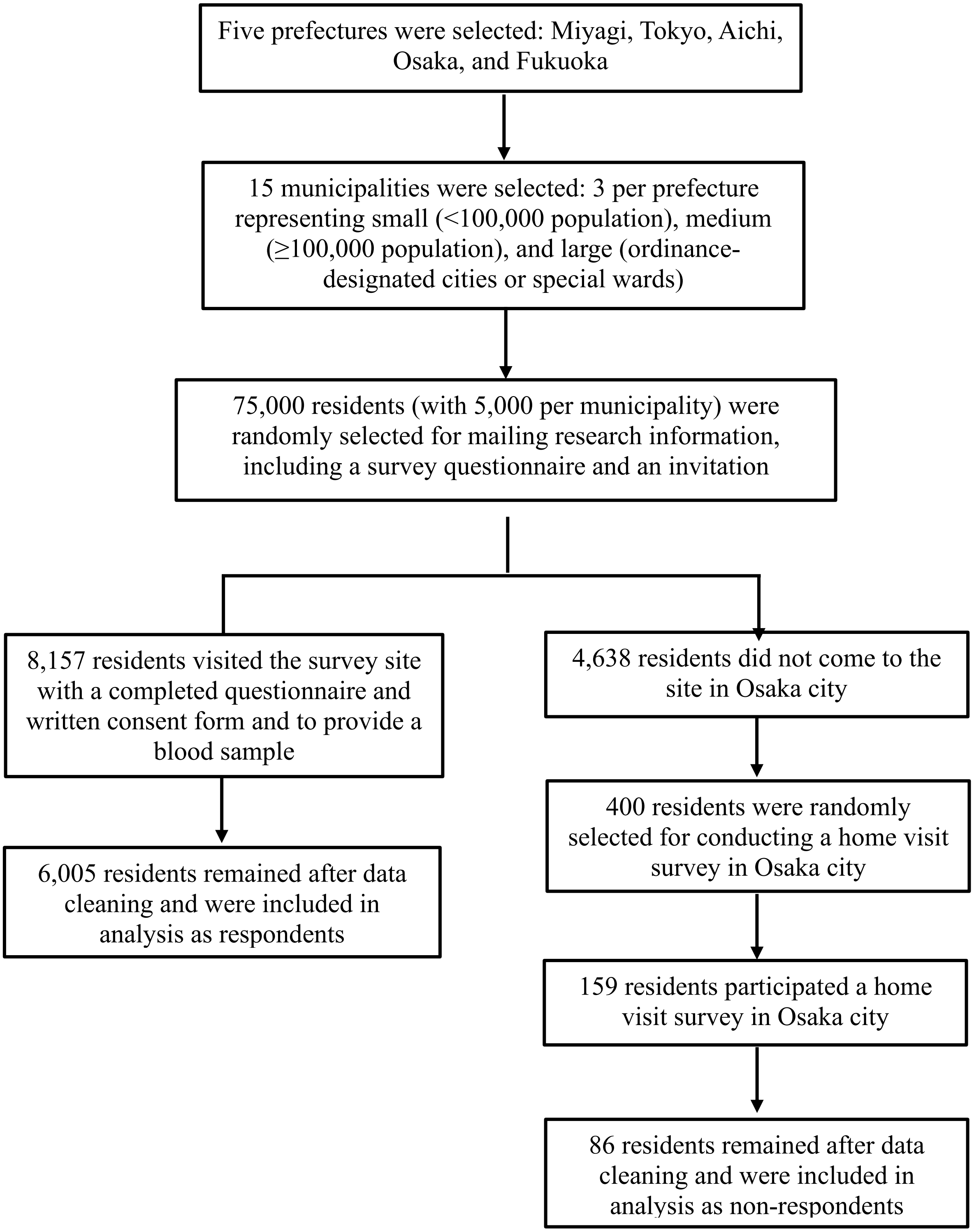

eFigure 2. Flowchart illustrating the sampling procedure for respondents (6,005) and non-respondents (86) of seroepidemiological survey

Sociodemographic factors

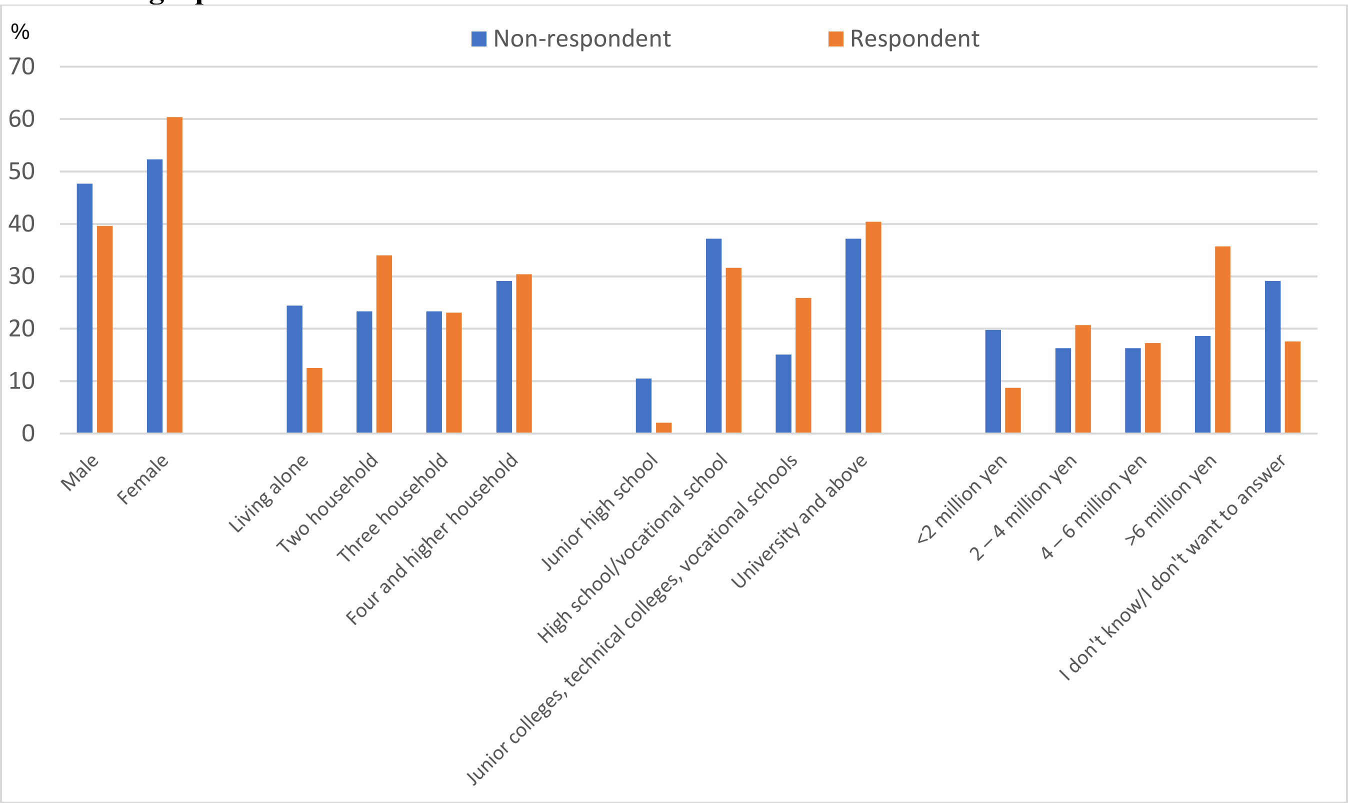

COVID-19 related factors

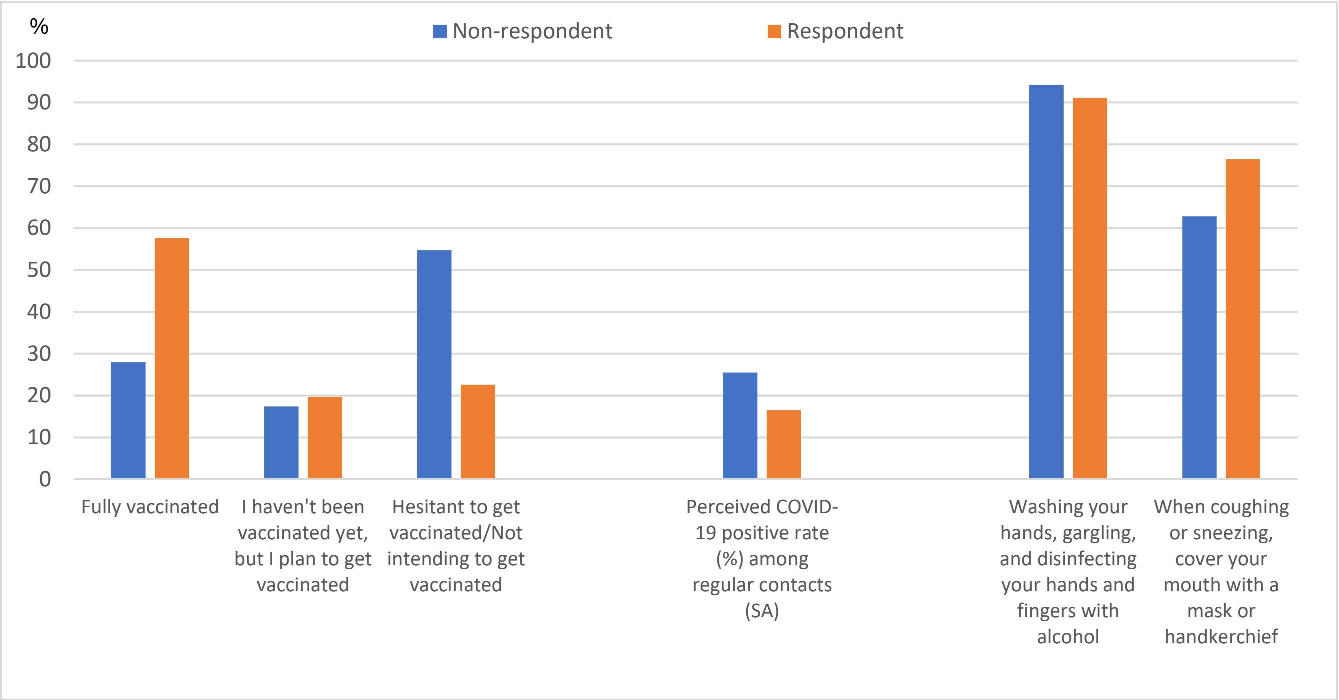

eFigure 3. Bar graph comparing the proportion of statistically significant factors among respondents and non-respondents in the seroepidemiological survey
